# Phytoagent Deoxyelephantopin and Its Derivative Inhibit Triple Negative Breast Cancer Cell Activity through ROS-Mediated Exosomal Activity and Protein Functions

**DOI:** 10.3389/fphar.2017.00398

**Published:** 2017-06-29

**Authors:** Jeng-Yuan Shiau, Yong-Qun Chang, Kyoko Nakagawa-Goto, Kuo-Hsiung Lee, Lie-Fen Shyur

**Affiliations:** ^1^Institute of Biotechnology, National Taiwan UniversityTaipei, Taiwan; ^2^Agricultural Biotechnology Research Center, Academia SinicaTaipei, Taiwan; ^3^Department of Biochemical Science and Technology, College of Life Science, National Taiwan UniversityTaipei, Taiwan; ^4^College of Medical, Pharmaceutical and Health Sciences, Kanazawa UniversityKanazawa, Japan; ^5^Natural Products Research Laboratories, Eshelman School of Pharmacy, University of North Carolina at Chapel Hill, Chapel HillNC, United States; ^6^Graduate Institute of Pharmacognosy, Taipei Medical UniversityTaipei, Taiwan

**Keywords:** breast cancer, sesquiterpene lactone, oxidative stress, exosomal proteome, cancer therapy

## Abstract

A novel plant sesquiterpene lactone derivative, DET derivative (DETD)-35, originating from parental deoxyelephantopin (DET) was previously observed to effectively suppress human triple negative breast cancer (TNBC) MDA-MB-231 cell activity and tumor growth in mice. In this study, the mechanisms underlying the activity of DETD-35 were elucidated. DET and DETD-35 induced reactive oxygen species (ROS) which caused structural damage and dysfunction of mitochondria and increased cytosolic calcium level, subsequently evoking exosome release from the cancer cells. Intriguingly, exosomes induced by both compounds had an atypical function. Cancer cell-derived exosomes commonly show metastatic potential, but upon DET/DETD-35 treatment exosomes showed anti-proliferative activity against MDA-MB-231 cells. Quantitative proteome analysis of TNBC cell-secreted exosomes showed that DET and DETD-35 attenuated the expression of proteins related to cell migration, cell adhesion, and angiogenesis. Furthermore, several exosomal proteins participating in biological mechanisms such as oxidative stress and decrease of transmembrane potential of mitochondria were found deregulated by treatment with either compound. Pretreatment with ROS scavenger, *N*-acetylcysteine, blockaded DET- or DETD-35-induced oxidative stress and calcium dependent exosome release mechanisms, and also reverted DET- or DETD-35-induced reprogramming exosomal protein expression profiles resulting in attenuation of exosomal toxicity against TNBC cell proliferation. In summary, this study shows that a plant-derived sesquiterpene lactone DET and its analog DETD-35 inhibitory TNBC cell activities through oxidative stress-induced cancer cell releasing exosomes in tandem with alteration of exosomal protein composition and functions. The findings of this study suggest that DETD-35 may be suitable for further development into an anti-TNBC drug.

## Introduction

Triple negative breast cancer (TNBC) is a breast cancer subtype lacking expression of estrogen receptor (ER), progesterone receptor (PR), and human epidermal growth factor receptor 2 (HER2) and accounts for about 15–25% of total breast cancer patients (Jamdade et al., 2015). TNBC commonly has a high recurrence rate in the first 3 years after diagnosis, worse prognosis, high risk of distant visceral metastasis, and is often observed in younger patients (Morris et al., 2007; Ovcaricek et al., 2011). Since the tumor cells are deficient in the necessary receptors, common hormone therapy or target therapies are inefficient, and therefore a combination of chemotherapy drugs are often used on TNBC patients; however, such a therapeutic approach cannot circumvent chemotherapy-induced drug resistance and adverse side effects in cancer patients (Chougule et al., 2011; von Minckwitz and Martin, 2012). Thus there is need to develop novel intervention approaches or therapeutic agents with minimal toxicities or better efficacy for TNBC patients.

Exosomes are nanoscale (40–100 nm diameter) membrane-bound vesicles specialized for long distance intercellular communication, facilitating the delivery of cargo proteins, mRNA, miRNA, etc. into target cells (Corrado et al., 2013). Exosomal proteomes from different origins contain a conserved set of common proteins for exosomal biogenesis, structure, and trafficking, and a specific subset of proteins associated with cell type-related functions (Raimondo et al., 2011; Choi et al., 2015). Cancer cell-derived exosomes can elicit pre-metastatic niche formation to promote cancer metastasis. For example, pancreatic cancer-derived exosomes promote liver metastasis by generating an immune cell infiltrated fibrotic microenvironment that favors metastasis (Zhang and Wang, 2015). In addition, cancer cells can impart an oncogenic environment by manipulating surrounding cells via exosomes (Melo et al., 2014). Deciphering a cell-type specific set of exosomal proteins may offer an ideal option for the development of exosome-based biomarkers and therapies. Intrinsically, exosomes possess the ability to cross biological barriers; therefore, exosome-encapsulated drug or bioactive natural product formulation are considered to be a versatile strategy for treating cancers or other inflammatory disorders (Zhuang et al., 2011; Haney et al., 2015; Ha et al., 2016).

Developing plant-derived phytocompounds as potential chemotherapeutic or chemopreventive agents for human cancers has generated a great deal of research interest. In our previous studies, we identified deoxyelephantopin (DET), a major germacranolide sesquiterpene lactone isolated from a perennial medicinal herb *Elephantopus scaber* L., which shows polypharmacological activities against TS/A(ER+) mammary tumor cell growth and metastasis. The anti-TS/A cancer cell activity of DET is through ROS/c-Jun N-terminal kinases (JNK)-mediated apoptosis, deregulation of nuclear factor kappa-light-chain-enhancer of activated B cells (NF-κB)/IκB kinase (IKK) pathways and ubiquitin-proteasome machinery, which impedes cancer cell motility by inhibiting calpain-mediated adhesion dynamics, and formation of centrosomal aggregates among others (Huang et al., 2010; Lee et al., 2010; Lee and Shyur, 2012). DET also showed pleiotropic function against lipopolysaccharide/D-galactosamine (LPS/D-GalN)-induced fulminant hepatitis by attenuating proinflammatory macrophage infiltration and cytokines, cyclooxygenase-2 (COX-2), and inducible nitric oxide synthase (iNOS) expression (Huang et al., 2013). However, relatively less inhibitory activity was found for human TNBC cell line, MDA-MB-231, a highly metastatic breast cancer type. Recently, we modified the DET structure to create novel DET derivatives (DETDs) by semi-organic synthesis, and obtained a number of bioactive DETDs. Among these derivatives, DETD-35 showed the best suppression of tumor growth and lung metastasis in MDA-MB-231 tumor-bearing NOD.CB17-*Prkdc*^scid^/NcrCrl (NOD/SCID) mice compared to the parental DET (Nakagawa-Goto et al., 2016). The underlying molecular mechanisms remains unexplored.

Cancer cell-derived exosomes have been demonstrated to play a role in promoting cancer cell invasiveness and metastasis, and activation of relevant oncogenic pathways (Higginbotham et al., 2011; Urbanelli et al., 2013; Ciardiello et al., 2016). Very recently, we have observed that DET and DETD-35 can induce ROS production in the MDA-MB-231 cells (Shiau et al., 2017). We thus hypothesized that there might be an association between ROS production and exosome release/activities, and these activities may be triggered by DET and/or DETD-35 as a mechanism for suppression of TNBC cell activities. Therefore, to shed light on the molecular mechanisms through which DET and DETD-35 suppress TNBC cell activity, in this study, we investigated whether TNBC cell-derived exosomes and their cargo proteins are affected by DET and/or DETD-35 treatment. We observed that both DET and DETD-35 can indeed significantly enhance MDA-MB-231 cell secretion of exosome vesicles into media. Of note, both compounds induced ROS production and calcium ion flux in cancer cell cytosols could impact exosome release. Unexpectedly, exosomes collected from DET- or DETD-35-treated cancer cells showed a significant inhibitory effect on the same recipient TNBC cells. A comparative exosome proteome study revealed a group of oxidative stress and mitochondrial function-related proteins which were commonly or differentially regulated by DET or DETD-35, revealing the novel molecular mechanisms of both compounds against TNBC cells.

## Materials and Methods

### Chemicals and Antibodies

3-(4,5-Dimethylthiazol-2-yl)-2,5-diphenyltetrazolium bromide (MTT), dimethyl sulfoxide (DMSO), ionomycin, 4,6-diamidino-2-phenylindole (DAPI), *N*-acetyl-L-cysteine (NAC), DL-dithiothreitol (DTT), 1,2-bis(2-aminophenoxy)ethane-*N,N,N*′,*N*′-tetraacetic acid tetrakis (acetoxymethyl ester) (BAPTA-AM), and iodoacetamide (IAA) were purchased from Sigma-Aldrich. Amplex Red acetlycholinesterase (AChE) assay kit, MitoProbe 1,1′,3,3,3′,3′-hexamethylindodicarbocyanine iodide [DiIC_1_(5)], and Fluo-4 AM were purchased from Thermo Fisher Scientific. DMEM, fetal bovine serum (FBS), and the antibiotics mixture (penicillin-streptomycin) were purchased from Invitrogen. Primary antibodies against voltage-dependent anion channel 1 (VDAC1), tumor susceptibility gene 101 protein (tsg101), CD9 and glyceraldehyde-3-phosphate dehydrogenase (GAPDH) (Santa Cruz Biotechnology), HSP27 (GeneTex), and β-actin (Chemicon, Millipore) were used.

### Preparation of DET and Its Derivative DETD-35

The extraction and identification of plant DET compound from the traditional medicinal herb *E. scaber* L. followed the protocol reported by Huang et al. (2010). The synthesis of DETD-35 followed the method described by Nakagawa-Goto et al. (2016). The chemical purity of DET and DETD-35 were >99% as judged by NMR spectrometry.

### Cell Culture

Human TNBC MDA-MB-231 cells obtained from ATCC, United States were grown in the manufacturers’ suggested medium supplemented with 10% FBS, 1 mM sodium pyruvate (Gibco) and 100 units/mL penicillin, and incubated in a humidified 5% CO_2_ incubator at 37°C.

### Isolation and Characterization of Exosomes

MDA-MB-231 cells (4 × 10^6^ cells/dish) were grown in a 15 cm dish using exosome-depleted medium and incubated overnight to allow cell adhesion. The medium was then replaced with fresh exosome-depleted medium, and subsequently TNBC cells were cultured for the indicated time periods (4, 8, and 12 h). Exosomes were collected from several runs of ultracentrifugation based on a published protocol with some modifications (Théry et al., 2006). Briefly, the culture medium was collected and centrifuged at 300 × *g* and 2000 × *g* for 10 min at 4°C to exclude dead cells. The supernatant was further centrifuged at 16500 × *g* for 30 min at 4°C to eliminate cell debris contamination. The exosomes were then pelleted through ultracentrifugation at 120,000 × *g* for 120 min at 4°C. The exosome pellet was washed using PBS buffer, and ultracentrifuged at 120,000 × *g* for 120 min again to remove the contaminating proteins. The exosome pellet was re-dissolved in the PBS buffer and stored at -80°C. Finally, the quantification of exosomes derived from TNBC cells was performed using Amplex Red acetlycholinesterase (AChE) assay kit according to the manufacturer’s protocol.

TNBC-secreted exosomes were further confirmed using transmission electron microscopy (TEM). Exosomes were fixed with 1% glutaraldehyde in 1× PBS for 10 min, and then the fixed sample was loaded on a carbon/formvar coated grid and dried on filter paper under vacuum for 20 min. The grids were washed with distilled water and negatively stained with 2% aqueous uranyl acetate for 30 s. Grids were air dried and then examined using TEM (FEI Tecnai G2 F20 S-TWIN FEGTEM).

### Cell Viability Assay

MDA-MB-231 cells (5 × 10^3^ cells/well) were plated in 96-well culture plates and incubated overnight at 37°C. The cells were treated with exosomes originating from vehicle- or compound-treated cells (0.5% DMSO, 11 μM DET, or 3 μM DETD-35) for 24 h. Cell growth was examined using MTT-based colorimetric assay as previously described (Chiang et al., 2005).

### Measurement of Mitochondrial Membrane Potential

Breast cancer cells (1.5 × 10^5^ cells/well) were seeded in 6-well culture plates overnight and treated with vehicle (DMSO, 0.5%), DET (11 μM), or DETD-35 (3 μM) for 2 h at 37°C. Then, 5 μL of 10 μM DiIC_1_(5) fluorescence dye was added into the treated cells and incubated for 30 min at 37°C. After washing cells with PBS, the mitochondrial membrane potential of treated cells was determined by flow cytometry.

### Determination of Intracellular Calcium Concentration

MDA-MB-231 cells (1.5 × 10^5^ cells/well) were grown in 6-well culture plates and incubated overnight to allow cell adhesion. The cells were treated with vehicle (DMSO, 0.5%), DET (11 μM), or DETD-35 (3 μM) for 2 h. Cells were washed with Hank’s buffered salt solution (HBSS) (Gibco) and then incubated in HBSS containing 2 μM Fluo-4 AM calcium indicator at 37°C for 30 min. The vehicle- or compound-treated cells were washed with HBSS and the positive control group consisted of cells treated with HBSS containing 4 μM ionomycin, a Ca^2+^ ionophore, for 5 min. Flow cytometry was used to measure the change of Fluo-4 AM fluorescence intensity in the treated cells.

### Protein Digestion and iTRAQ Labeling

Exosome pellets from vehicle- or compound-treated cells were lysed with 8 M urea in 50 mM Tris buffer (pH 8.5). Protein concentration was measured by Pierce 660 nm protein assay (Thermo Scientific, Rockford, IL, United StatesA) according to the manufacturer’s protocol. Twenty-five micrograms of exosome samples were reduced with 10 mM DL-dithiothreitol (DTT) in 8 M urea in 50 mM Tris buffer (pH 8.5) at 37°C for 1 h and subsequently alkylated with 50 mM iodoacetamide (IAA) in 8 M urea in 50 mM Tris buffer (pH 8.5) at room temperature (RT) for 30 min in the dark. The reaction was stopped with 100 mM DTT in 8 M urea in 50 mM Tris buffer (pH 8.5), and the urea was diluted to a concentration of 4 M urea with 50 mM Tris buffer (pH 8.5). The Lys-C protease (1:200) (Wako) was added and incubated at RT for 4 h. Finally, the solution was diluted to 0.8 M urea concentration with 50 mM Tris buffer (pH 8.0), and then trypsin (1:50) (Promega) was added and the solution was incubated overnight at 37°C. An Oasis HLB column (Waters Corporation) was employed to remove detergents or salts from the solution of digested proteins and dried in a SpeedVac. Each digested sample was labeled with 4-plex iTRAQ reagent (AB SCIEX, Foster City, CA, United States) according to the manufacturer’s protocol. Specific labeling was performed as 114, 115, and 116 isobaric tag 4-plex iTRAQ reagents for vehicle control, DET, and DETD-35 exosomal samples, respectively. Three iTRAQ-labeled exosomal samples were combined and dried using a SpeedVac.

### Strong Cation Exchange Fractionation and LC-MS/MS Analysis

The combined samples were resuspended in 10 mM KH_2_PO_4_, pH 2.65, 25% acetonitrile (Buffer A) and then loaded into the Strong Cation Exchange (SCX) column (Polysulfoethyl A, 4.6 × 200 mm, 5 μm polysulfoethyl aspartamide beads, 300 Å pore size) (Poly LC Inc.) at a flow rate of 1 ml/min. The gradient was started with 0% Buffer B (1 M KCl in Buffer A, pH 2.65), then increased to 15% Buffer B at 20 min, to 30% Buffer B for 10 min, to 50% Buffer B for 5 min and held at 50% Buffer B for 5 min, and finally 1 min to 100% Buffer B. The fraction was collected every 30 sec, and subsequently desalted with Oasis HLB column and dried using a SpeedVac.

The LC-nESI-Q Exactive mass spectrometer model from Thermo Fisher Scientific coupled with an on-line nanoUHPLC (Dionex UltiMate 3000 Binary RSLCnano) was used for protein identification and analysis. An Acclaim PepMap 100 C18 trap column (75 μm × 2.0 cm, 3 μm, 100 Å, Thermo Fisher Scientific) and an Acclaim PepMap RSLC C18 nano LC column (75 μm × 15 cm, 2 μm, 100 Å) were utilized to deliver solvent and separate tryptic peptides with a linear gradient from 3 to 30% of acetonitrile in 0.1% (v/v) formic acid for 3 h at flow rate of 300 nL/min. The acquisition of the MS data was performed in data dependent mode with a full MS scan followed by 10 MS/MS scans of the top 10 precursor ions from the MS scan. The MS scan was performed with a resolving power of 70,000 over the mass-to-charge (m/z) range 380 to 1800 and dynamic exclusion enabled. The data dependent MS/MS acquisitions were performed with: 2 m/z isolation window, 27 NCE, and 17,500 resolving power.

### Protein Identification and Quantitation

Peptide and protein identification was performed using the Proteome Discoverer software (v.1.4.1.14., Thermo Fisher Scientific) with SEQUEST and MASCOT search algorithms (Matrix Science) against a Swiss-Prot human protein database of Human uniprot 148,986 entries. The parameters for database searches were set as follows: full trypsin digestion with two maximum missed cleavage sites, precursor mass tolerance of 10 ppm, fragment mass tolerance of 0.02 Da, dynamic modifications of oxidation at methionine (M) residues, and static modifications of carbamidomethylation at cysteine (C) residues, iTRAQ 4plex at lysine residues and N-terminal proteolytic peptides. The identified peptides were validated using Percolator algorithm against the decoy database search which rescored peptide spectrum matches (PSM) by *q*-values and posterior error probabilities. All the peptides were filtered with a *q*-value threshold of 1% false discovery rate (FDR), with the identified protein having a minimum of two unique peptides. For quantitative analysis, the relative abundance of each protein present in two biological replicates was calculated based on the iTRAQ reporter ion ratios of 115/114 and 116/114 found at the peptide level. The *Z*-score cutoff of ±1.96σ (representing 95% confidence level) was set as a threshold for defining differentially expressed proteins (Berard et al., 2012).

### Bioinformatics Analysis

Molecular functions of the differentially expressed proteins from both compound treatments relative to vehicle control were analyzed on the Gene Ontology (GO) search through Proteome Discoverer application to ProteinCenter database. GO biological process annotation of the up- and down-regulated proteome observed in compound treatment was carried out using DAVID bioinformatic Resources 6.8. The significantly enriched biological processes of the up- and down-regulated protein expressions in the exosomal proteome were picked out, and the threshold of statistical significance was set as the –log(*p*-value) value of 1.3 (*P* < 0.05). The Ingenuity Pathway Analysis (IPA) database search tool (Ingenuity Systems) was used to further analyze the differentially expressed proteins responsive to both compound treatments involved in biological mechanisms that relate to canonical pathways and toxicity. The -log(*p*-value) of 1.3 was considered statistically significant in the canonical pathway and toxicity list analysis of IPA.

### Western Blot Analysis

The collected TNBC cells were subjected to lysis by radio-immunoprecipitation assay (RIPA) lysis buffer (Santa Cruz Biotechnology, United States). Protein concentration was measured by Pierce 660 nm protein assay (Thermo Scientific, Rockford, IL, United States) according to the manufacturer’s protocol. Protein samples were separated by 10 or 12.5% SDS-PAGE, and subjected to transblotting analysis according to a method published elsewhere (Lee et al., 2010). Enhanced chemiluminescent detection reagents (Amersham; Thermo Scientific) were employed to visualize the reactive protein bands by exposure to chemiluminescence light film (BioMax; Kodak Co.). Quantification of the reactive protein band expression used ImageJ software.

### Immunofluorescence Cell Staining

MDA-MB-231 cells (2 × 10^4^ cells/well) were seeded on glass slips in 24-well culture plates overnight, and then treated with vehicle (DMSO, 0.5%), DET (11 μM), or DETD-35 (3 μM) for 24 h. The fix and block steps of treated cells followed the method previously described (Lee and Shyur, 2012). After washing with PBS, cells on glass slips were incubated with primary antibody VDAC1 in blocking buffer (1:100) at 4°C for 18 h, and then washed and stained with FITC conjugated secondary antibody (1:200) at RT for 3 h (Jackson ImmunoResearch Laboratories). The nuclei region of treated cells was stained with DAPI (Sigma–Aldrich), and the cells were mounted onto a glass slide with Gold Antifade Reagent (ProLong). The Zeiss LSM 780 plus Elyra confocal microscope was used to visualize and capture the stained cells.

### Transmission Electron Microscopy

MDA-MB-231 cells were prefixed with 0.1 M cacodylate buffer containing 2.5% glutaraldehyde and 0.1% tannic acid for 30 min at RT, and washed with PBS. Post-fixing was carried out using 1% osmium tetroxide in 0.1 M cacodylate buffer at RT for 30 min, and then washed and dehydrated through a graded series (30–100%) of ethanol. The cells were embedded in Spurr’s resin (EMS), and polymerized at 70°C for 48 h. The polymerized samples were sectioned and stained with uranyl acetate and lead citrate, and then visualized by electron microscopy (FEI Tecnai G2 F20 S-TWIN FEGTEM).

### Statistical Analysis

All data are presented as means ± standard deviation (SD). Statistical analysis of experimental results was carried out using the SAS program (SAS Institute), and significant differences between different treatment groups were examined using ANOVA. *P*-values of less than 0.05 were regarded as a statistically significant.

## Results

### DET and DETD-35 Caused Structural Damage and Dysfunction of Mitochondria in MDA-MB-231 Cells

**FIGURE 1:**
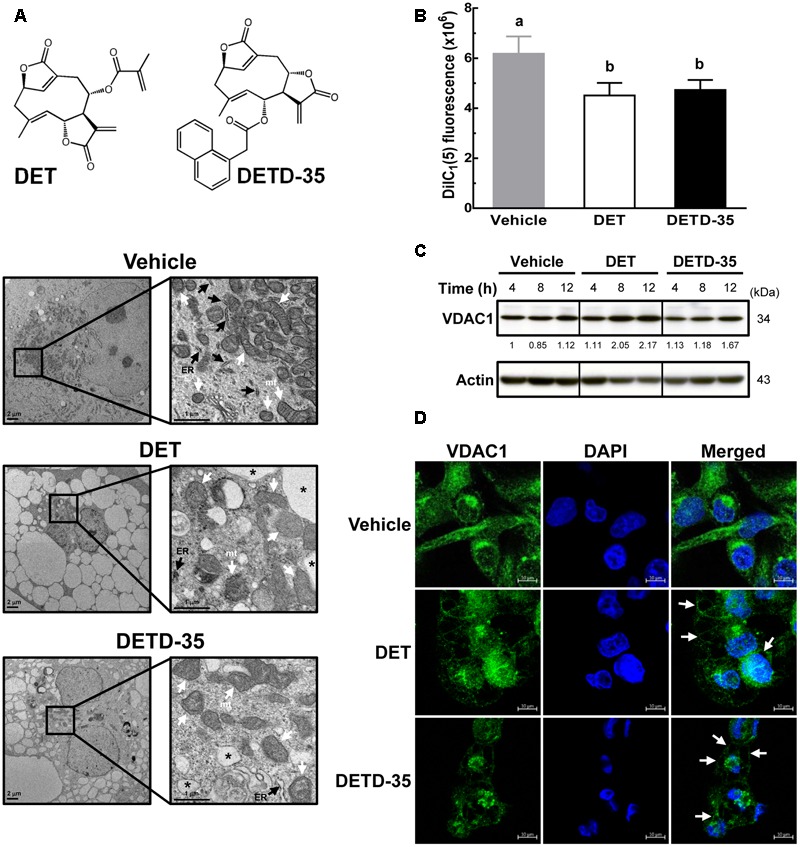
Deoxyelephantopin (DET) and DET derivative (DETD)-35 induced morphological change and challenged structural integrity of mitochondria in MDA-MB-231 cells. **(A)** MDA-MB-231 cells were treated with vehicle (0.5% DMSO) or IC_50_ concentrations for inhibiting MDA-MB-231 cells of DET (11 μM) and DETD-35 (3 μM) for 24 h, and the morphological changes of cancer cells were examined by transmission electron microscopy (TEM) imaging (10,000× magnification). The ER, mitochondria (mt), and cytoplasmic vacuoles are indicated by black arrowheads, white arrowheads, and asterisks, respectively. **(B)** MDA-MB-231 cells were treated with vehicle (0.5% DMSO), 11 μM DET, and 3 μM DETD-35 for 2 h. The vehicle– and compound–treated cells were labeled with DiIC_1_(5) (10 μM) fluorescence dye for 30 min, and mitochondrial membrane potential of the treated cells was determined using flow cytometry. Data are mean ± SEM, *n* = 3. Different letters indicate significant differences (one-way ANOVA, *P* < 0.05). **(C)** Western blotting of VDAC1 protein in MDA-MB-231 cells treated with vehicle (0.5% DMSO), 11 μM DET, or 3 μM DETD-35 for 4, 8, and 12 h. Quantification of the corresponding protein bands was performed using ImageJ software. β-actin was used as a loading control. **(D)** Immunofluorescence analysis of MDA-MB-231 cells treated with vehicle (0.5% DMSO), DET (11 μM), or DETD-35 (3 μM) for 24 h. The treated cells were fixed using 100% ice-cold methanol, and stained with VDAC1 (green), and DAPI (blue) to visualize mitochondrial structures and nuclei. White arrowheads indicate DET– and DETD-35–induced cytoplasmic vacuoles.

According to our previous investigation, DETD-35 has a more potent anti-proliferative effect than the parental DET, showing an approximate 3.5-fold decrease (11 μM vs. 3 μM) in IC_50_ value in the human TNBC cell line, MDA-MB-231 (Shiau et al., 2017). In this study, we treated TNBC cells with the IC_50_ concentration of DET (11 μM) and DETD-35 (3 μM) for 24 h and examined the phenotypic changes using TEM. As shown in **Figure 1A**, the intact rough endoplasmic reticulum (RER) structures made by the ribosomes decorating the RER membrane were only seen in the vehicle-treated cells, and were not observed treatment with either compound. Both compounds significantly induced massive cytoplasmic vacuole (asterisks) formation in MDA-MB-231 cells. The structural integrity of mitochondria (mt) was also damaged in compound-treated cells compared to the vehicle control. We further evaluated whether both DET- and DETD-35-induced morphological damage in mitochondria caused mitochondrial dysfunction in the treated TNBC cells. The status of the mitochondrial membrane potential was first determined using MitoProbe DiIC_1_(5) fluorescent dye in the TNBC cells treated for 2 h. A loss of mitochondrial membrane potential in cells by either DET (27%) or DETD-35 (23.5%) treatment relative to vehicle-treated cells (100%) was indeed observed (**Figure 1B**). Of note, at 12 h treatment with DET, protein expression level of voltage-dependent anion channel 1 (VDAC1), a mitochondrial metabolite transporter critical in the activation of the mitochondria-mediated apoptotic process had increased about 2-fold; DETD-35 treatment for the same time period induced a 1.67-fold increase, both relative to the vehicle control (**Figure 1C**). Confocal microscopy was further used to examine the immunofluorescence staining of VDAC1 in the 24-h treated MDA-MB-231 cells. Positive staining of VDAC1 protein (green) was observed mainly distributed in the cytoplasm of vehicle-treated cells; however, in DET- and DETD-35-treated cells, the VDAC1 protein appeared at the membrane surrounding the expanded vacuoles in the cytosol or accumulated in the perinuclear region and less VDAC1 expression was also observed in DETD-35-treated cells (**Figure 1D**). The data suggest that both DET- and DETD-35-induced vacuole formation might be in part derived from mitochondria that might cause mitochondrial dysfunction in the human TNBC cells.

### DET and DETD-35 Increased Cytosolic Calcium Concentration in TNBC Cells

**FIGURE 2:**
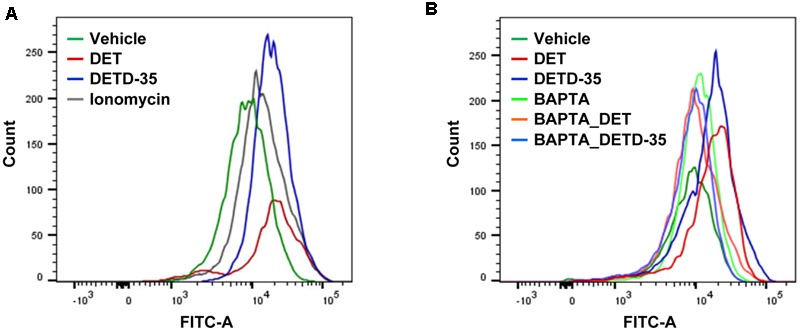
Deoxyelephantopin and DETD-35 induced the calcium ion levels in TNBC cells. **(A)** MDA-MB-231 cells were treated with vehicle (0.5% DMSO), DET (11 μM), or DETD-35 (3 μM) for 2 h. In parallel, TNBC cells were treated with ionomycin (4 μM) for 5 min as a positive control. Cytosolic free calcium level was determined with Fluo-4 Ca^2+^ fluorescent dye in the treated cells using flow cytometry. **(B)** MDA-MB-231 cells were pre-treated with 20 μM calcium chelator BAPTA-AM for 30 min, and then treated with vehicle or both compounds for 2 h. Detection of cytosolic free calcium level in the treated cells used flow cytometry.

Deregulation of intracellular calcium homeostasis is known to be one of the features of mitochondrial dysfunction in cells (Boland et al., 2013). Next, we examined whether both DET and DETD-35 treatment can interfere with intracellular calcium level in TNBC cells. The calcium (Ca^2+^) sensitive fluorescent dye, Fluo-4 acetoxymethyl (AM) and flow cytometry were employed to analyze vehicle- or compound-treated MDA-MB-231 cells to detect the change of cytosolic free Ca^2+^ level. The result, shown in **Figure 2A**, shows that the mean intensity of Fluo-4 dye in DET- and DETD-35-treated TNBC cells was significantly induced 2.4- and 2.0-fold, respectively, compared to vehicle-treated cells. The increase in Fluo-4 fluorescence intensity (∼1.8-fold) was also observed in the cancer cells treated with calcium ionophore ionomycin, used as a positive control in this experiment. On the other hand, pretreatment for 30 min with 20 μM calcium chelator, 1,2-bis(2-aminophenoxy)ethane-*N,N,N*′,*N*′-tetraacetic acid tetrakis (BAPTA-AM), before either DET or DETD-35 treatment effectively diminished the increase of Fluo-4 fluorescence intensity in compound-treated cells and the Ca^2+^ levels were similar to the vehicle-treated cells (**Figure 2B**). These data indicate that both compounds significantly promoted intracellular Ca^2+^ content, which may be because of their damaging effect on calcium storage organelles, such as the mitochondria and endoplasmic reticulum (ER) in the TNBC cells.

### DET and DETD-35 Promoted Ca^2+^-Dependent Release of Exosomes from TNBC Cells

**FIGURE 3:**
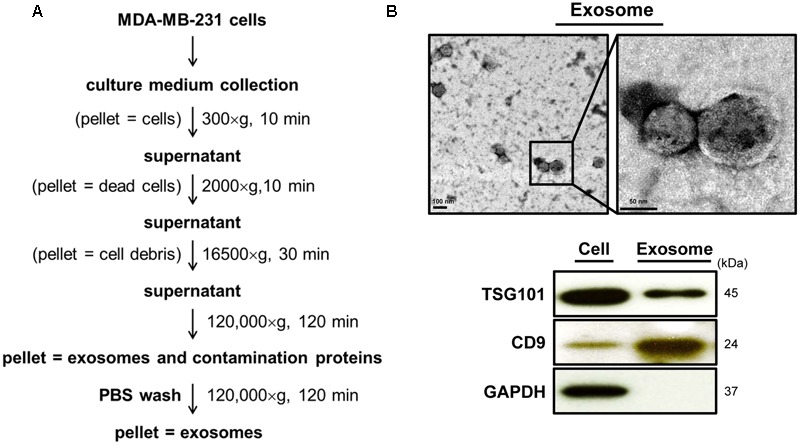
Characterization of the quality of MDA-MB-231 cell-derived exosomes. **(A)** Flow chart of cell-derived exosome isolation and purification from culture medium by differential centrifugation. **(B)** Purified exosomes from the 24-h vehicle–treated TNBC cells were examined using transmission electron microscopy (TEM) imaging (9,900× and 19,500× magnification). Immunoblotting of exosomal marker proteins, TSG101 and CD9, from the same batch of exosomes. GAPDH was used as a positive control of cytosolic proteins.

**FIGURE 4:**
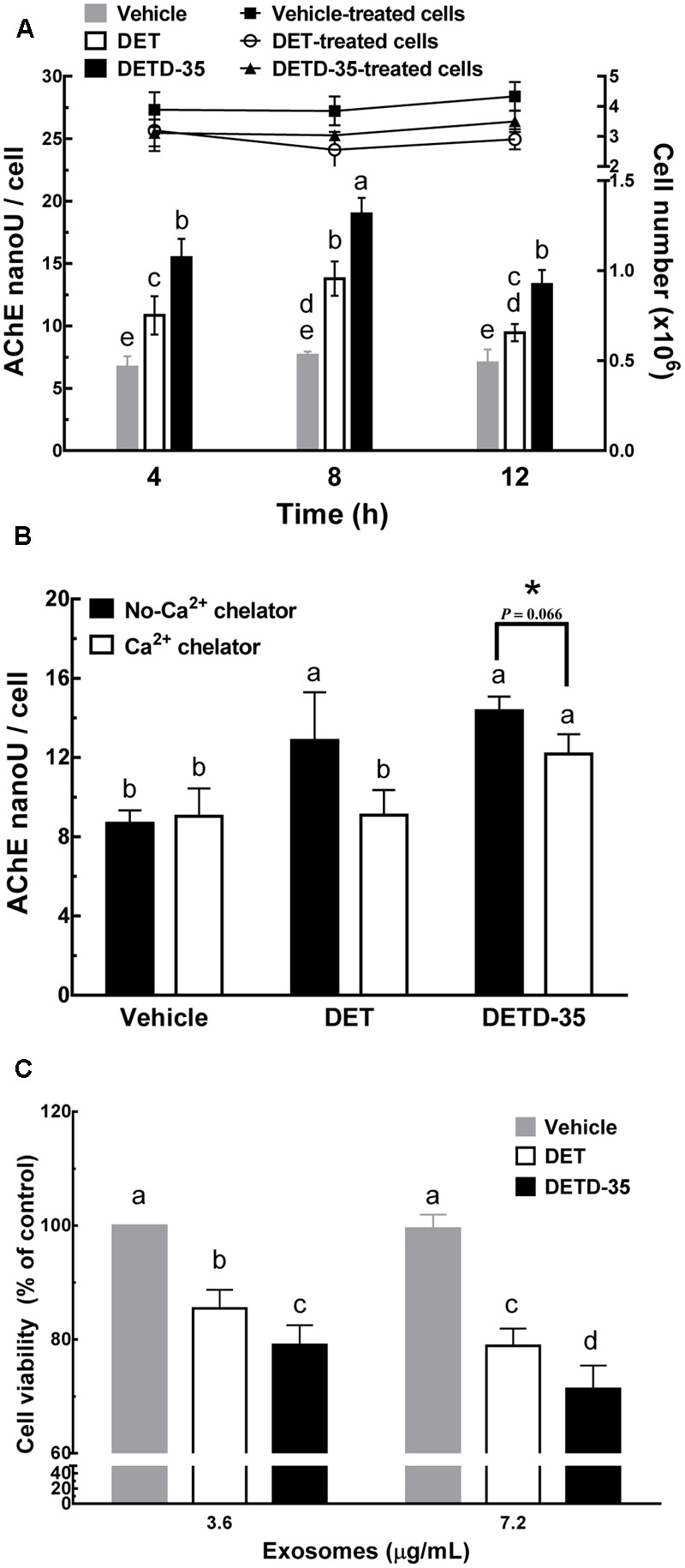
Deoxyelephantopin and DETD-35 promoted a time-dependent release of TNBC cell-derived exosomes. **(A)** MDA-MB-231 cells were treated with vehicle (0.5% DMSO), DET (11 μM), and DETD-35 (3 μM) at the indicated time periods (4, 8, and 12 h), and then the TNBC-secreted exosomes were collected from culture media using differential centrifugation. The activity of acetylcholinesterase (AChE), an exosome marker protein, was determined using fluorescence microplate reader. Data are mean ± SEM, *n* = 3. **(B)** MDA-MB-231 cells were pre-treated with 20 μM calcium chelator BAPTA-AM for 30 min, and then treated with either vehicle, DET or DETD-35 for 8 h. The release of treated cell-derived exosomes was separated by differential centrifugation. Data are mean ± SEM, *n* = 3. **(C)** The exosomes (3.6 and 7.2 μg/mL, respectively) collected from the culture medium of vehicle–, DET–, or DETD-35–treated cells) were added into the freshly cultured MDA-MB-231 cells for 24 h, and the TNBC cell viability was determined using MTT assay. Different letters indicate significant differences (one-way ANOVA, *P* < 0.05). The difference of DETD-35–treated cells with or without BAPTA-AM pretreatment presented in **(B)** was further analyzed by student *t*-test and labeled with asterisk.

Several articles have reported that the increase in intracellular calcium level in mammalian cells is positively correlated to the release of cell-derived exosomes (Savina et al., 2003; Urbanelli et al., 2013). We thus further evaluated whether DET or DETD-35 treatment can affect exosome release in TNBC cells using a differential ultracentrifugation approach adapted from a published protocol with some modifications (Théry et al., 2006) (**Figure 3A**). The quality of collected exosomes from the culture medium of TNBC cells was examined using TEM and immunoblotting of exosomal marker proteins, including TSG101 and CD9. As shown in **Figure 3B**, the diameter range of TNBC cell-secreted exosomes was between 50 and 100 nm which perfectly matched with typical exosome size range (40–100 nm diameter) (Simons and Raposo, 2009). The western blotting data showed that tumor susceptibility gene 101 protein (TSG101), a commonly used exosomal marker protein was indeed detected in the exosomes. A significant amount of TSG101 was also detected in the cytosolic protein fraction, as this protein is also a member of endosomal sorting complex required for transport (ESCRT) complex involved in the biogenesis of multivesicular bodies (MVBs) in the cells (Urbanelli et al., 2013). The representative exosomal marker protein CD9 was detected at the highest level in the TNBC cell-derived exosomes, while GAPDH, an abundant cytoplasmic protein, was only found in TNBC cell protein lysates, indicating high purity of collected exosomes secreted from TNBC cells. Next, we examined whether both DET and DETD-35 treatments influence the exosome release in MDA-MB-231 cells. Several batches of TNBC cell-derived exosomes were collected after treatment with vehicle control, DET (11 μM), and DETD-35 (3 μM) at the indicated times, and the total exosomal proteins were extracted and measured for the enzymatic activity of acetylcholinesterase (AChE), an exosomal marker protein. Surprisingly, both DET and DETD-35 treatment enhanced exosome release into the culture medium with the most significant induction revealed at the 8 h treatment compared to vehicle-treated cells (**Figure 4A**). The cell populations were similar in vehicle- or compound-treated cells. In view of such important phenomena being observed by DET/DETD-35 treatment, we further examined whether it was through Ca^2+^-dependent pathway. We designed a calcium chelator BAPTA-AM pretreatment experiment (for 30 min) prior to compound treatment (for 8 h) and quantified the released exosome amounts by measuring AChE activity. BAPTA-AM significantly prevented exosome release in the DET-treated cells (*P* < 0.05), and the Ca^2+^ chelator partially inhibited the effect of DETD-35 on exosome release (*P* < 0.066) (**Figure 4B**). These results indicated that exosome secretion stimulated by either compound is involved in the calcium-dependent mechanism. The role of exosomes in affecting the recipient or neighboring cell activity has been reported (O’Brien et al., 2013; Harris et al., 2015). We further investigated whether the secreted exosomes isolated from DET- or DETD-35-treated cancer cells may have an effect on TNBC cell activity, as a recipient. We collected the TNBC cell-derived exosomes with the highest secretion at 8 h vehicle or compound treatment, and added 3.6 and 7.2 μg/mL exosomes, respectively, to a culture medium of fresh MDA-MB-231 cells and incubated for 24 h. The TNBC cell viability was then evaluated by MTT assay. Strikingly, the exosomes secreted from the parental TNBC cells treated with DET or DETD-35 significantly inhibited the viability of recipient MDA-MB-231 cells dose-dependently compared to the exosomes collected from vehicle control cells (**Figure 4C**). The DETD-35-induced released exosomes showed more MDA-MB-231 cell proliferation inhibition activity than DET-induced exosomes (21–29% vs. 15–22% inhibition) (*P* < 0.05) in a dose-dependent manner. Together, these results indicate that both DET and DETD-35 promoted release of exosomes from TNBC cells is directly correlated to their effect on increasing intracellular Ca^2+^ levels in the cancer cells.

### Comparative Exosomal Proteomes Analysis

**FIGURE 5:**
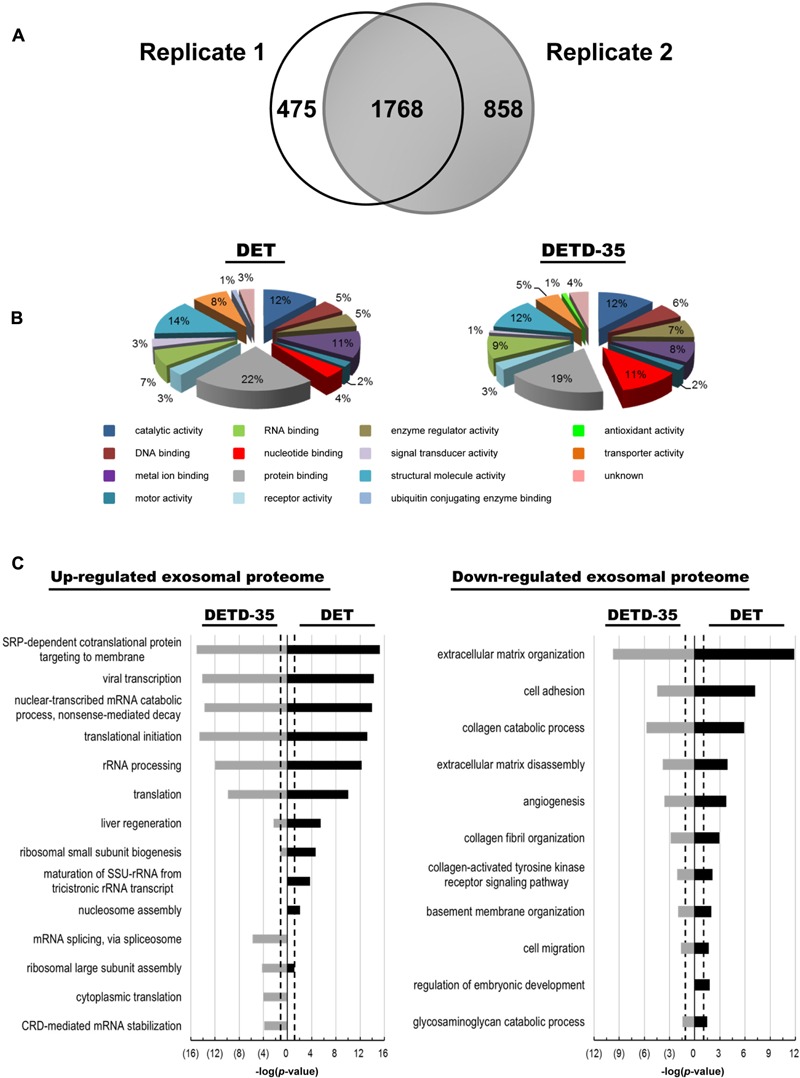
Comparison of exosomal proteome originated from DET– and DETD-35–treated MDA-MB-231 cells. **(A)** Venn diagram showing the overlapping and unique exosomal proteins between the two biological replicates in DET– or ETD-35–treated TNBC cells. **(B)** Molecular functions of significantly expressed compound-responsive exosomal proteins were classified based on the gene ontology (GO) categorization. **(C)** The significantly differential expression of exosomal proteins responsive to both compound treatments were clustered into groups according to the GO biological processes terms by DAVID Bioinformatics tools. The top 10 significantly enriched biological processes of the up- and down-regulated protein expressions in the exosomal proteome were selected out, and ranked on the basis of based on –log(*p*-value) value high than 1.3 (*P* < 0.05). The black dotted line indicates –log(*p*-value) value of 1.3 as the threshold for statistical significance.

Next, we used a mass spectrometry (MS)-based quantitative proteomics approach coupled with Ingenuity Pathway Analysis (IPA) database analysis to investigate whether the exosomes produced from compound-treated cells act as a deregulator against TNBC cell activity by changing their vesicle protein content or function. The differentially expressed exosomal proteins responsive to DET or DETD-35 treatment were analyzed at 8 h by isobaric tags for relative and absolute quantitation (iTRAQ)-based comparative exosomal proteomics. Based on at least two unique peptides and 1% FDR, we quantitatively identified a total of 2243 and 2626 proteins, respectively, in two biological replicates (Supplementary Tables S1, S2). Among them, 1768 proteins were commonly present in these two biological replicates (**Figure 5A** and Supplementary Table S3). The differentially expressed proteins in both compound treatments relative to vehicle control were selected based on *Z*-score cutoff of ±1.96σ (representing 95% confidence level) (Berard et al., 2012). The significantly up- and down-regulated exosomal proteins responsive to compound treatment were calculated and determined as 67 and 27 proteins for DET, and 71 and 28 proteins for DETD-35, respectively (Supplementary Tables S4, S5). Furthermore, the molecular functions of up- and down-regulated exosomal proteins observed in both compound treatments were identified based on a GO search. The significantly expressed exosomal proteins were mostly classified into the protein binding, structural molecule activity, catalytic activity, and metal ion binding categories (**Figure 5B**). The GO biological process annotation of the compound-responsive exosomal proteome was performed by the “database for annotation, visualization and integrated discovery” (DAVID) bioinformatic tool. All compound-responsive exosomal proteins were further classified into groups based on similar annotation terms describing the biological processes. The significant biological process of each group was picked out based on -log(*p*-value) of 1.3 (representing *P* < 0.05) as the threshold for statistical significance. As shown in **Figure 5C**, the top 6 significantly enriched biological processes of the up-regulated exosomal proteins responsive to DET or DETD-35 treatments were similar, such as SRP-dependent cotranslational protein targeting to membrane, viral transcription, nuclear-transcribed mRNA catabolic process, translational initiation, rRNA processing, and translation. However, unique biological processes of exosomal proteins were also seen in the two treatment groups. Nucleosome assembly, maturation of small subunit ribosomal RNA (SSU-rRNA) from tricistronic rRNA transcript, ribosomal small subunit biogenesis, and liver regeneration were specific to DET treatment, and coding region determinant (CRD)-mediated mRNA stabilization, cytoplasmic translation, ribosomal large subunit assembly, and mRNA splicing via spliceosome were specific to DETD-35 treatment. Conversely, the top 10 enriched biological processes of down-regulated exosomal proteins responsive to both compounds were quite focused on cell migration, basement membrane (BM) organization, collagen-activated tyrosine kinase receptor signaling pathway, collagen fibril organization, angiogenesis, extracellular matrix disassembly and organization, collagen catabolic process, and cell adhesion. Specifically, the protein expression of laminin subunit alpha-5 and subunit gamma-1, collagen alpha-1(V) chain involved in cell migration, collagen alpha-2(IV) chain, collagen alpha-1(XVIII) chain, lactadherin, and BM-specific heparin sulfate proteoglycan core protein for angiogenesis, and tubulointerstitial nephritis antigen-like, collagen alpha-1(XII) chain, lysyl oxidase homolog 2, epidermal growth factor (EGF)-like repeat and discoidin I-like domain-containing protein 3, and laminin subunit alpha-2 for cell adhesion activity were attenuated.

### Analysis of Differentially Expressed Proteins in Exosomes

**FIGURE 6:**
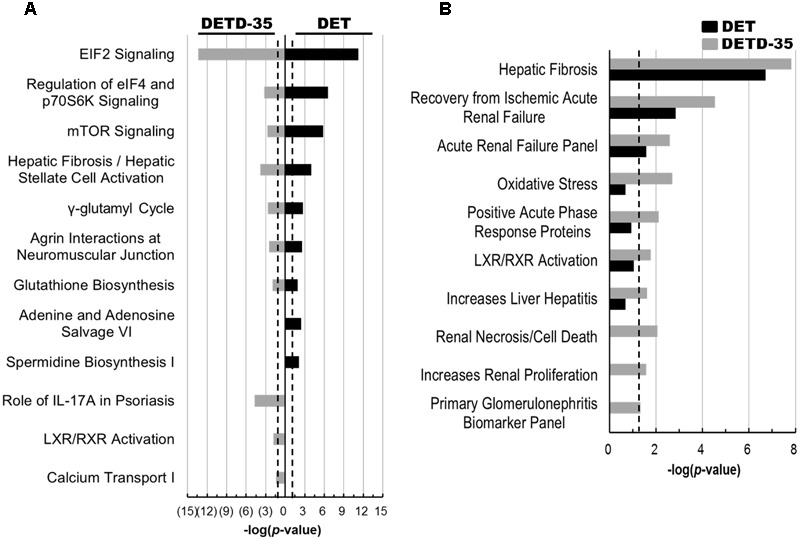
Canonical pathways and toxicity lists of differentially expressed compound-responsive exosomal proteins generated by IPA database analysis. The significant canonical pathways **(A)** and toxicological events **(B)** of the differentially expressed exosomal proteins responsive to both compound treatments were performed using IPA database analysis. The –log(*p*-value) of 1.3 (*P* < 0.05) was considered as statistical significance in IPA analysis. The black dotted line indicates –log(*p*-value) value of 1.3 as the threshold.

Next, the IPA database was employed to analyze the differentially expressed exosomal proteins involved in biological mechanisms that relate to canonical pathways and toxicity with the -log(*p*-value) of 1.3 (*P* < 0.05) set out as statistical significance. We observed that several exosomal proteins responsive to DET and DETD-35 treatment are commonly involved in mediation or regulation of eukaryotic initiation factor 2 (EIF2) signaling, eukaryotic initiation factor 4 (eIF4) and p70S6K signaling, mechanistic target of rapamycin (mTOR) signaling, hepatic fibrosis/hepatic stellate cell activation, γ-glutamyl cycle, and glutathione biosynthesis (**Figure 6A**). Nevertheless, specific canonical pathways unique to either compound were also found, for example, adenine and adenosine salvage VI and spermidine biosynthesis I specific for DET treatment, and role of interleukin 17A (IL-17A) in psoriasis, liver X receptor/retinoid X receptor (LXR/RXR) activation, and calcium transport I specific for DETD-35 treatment (**Figure 6A**). In the IPA toxicity list analysis, hepatic fibrosis, recovery from ischemic acute renal failure, and acute renal failure panel were significantly observed for both compound treatments (**Figure 6B**). Furthermore, DETD-35 responsive exosomal proteome was also implicated in some of the biological mechanisms related to toxicity such as oxidative stress, positive acute phase response proteins, LXR/RXR activation, increase liver hepatitis, renal necrosis/cell death, and primary glomerulonephritis biomarker panel (*P* < 0.05) (**Figure 6B** and Supplementary Tables S6, S7). Although these toxicity functions were also observed for DETD-35, in the toxicity list result of DET responsive exosomal proteome, the *P-*value was higher than 0.05 (*P* = 0.09–0.21). Together, the particular toxicity functions, (i.e., decrease of transmembrane potential of mitochondria and oxidative stress) observed in the proteomics study, lend strong support to the DET-/DETD-35-induced mitochondrial dysfunction characterized in this study, and the ROS-mediated cytotoxicity and programmed cell death against breast cancer cells or melanoma cells induced by DET/DETD-35 reported in our previous studies (Lee and Shyur, 2012; Feng et al., 2016; Nakagawa-Goto et al., 2016).

### ROS Plays a Role in DET/DETD-35 Regulated Exosome Release and Anti-TNBC Cell Proliferation

**FIGURE 7:**
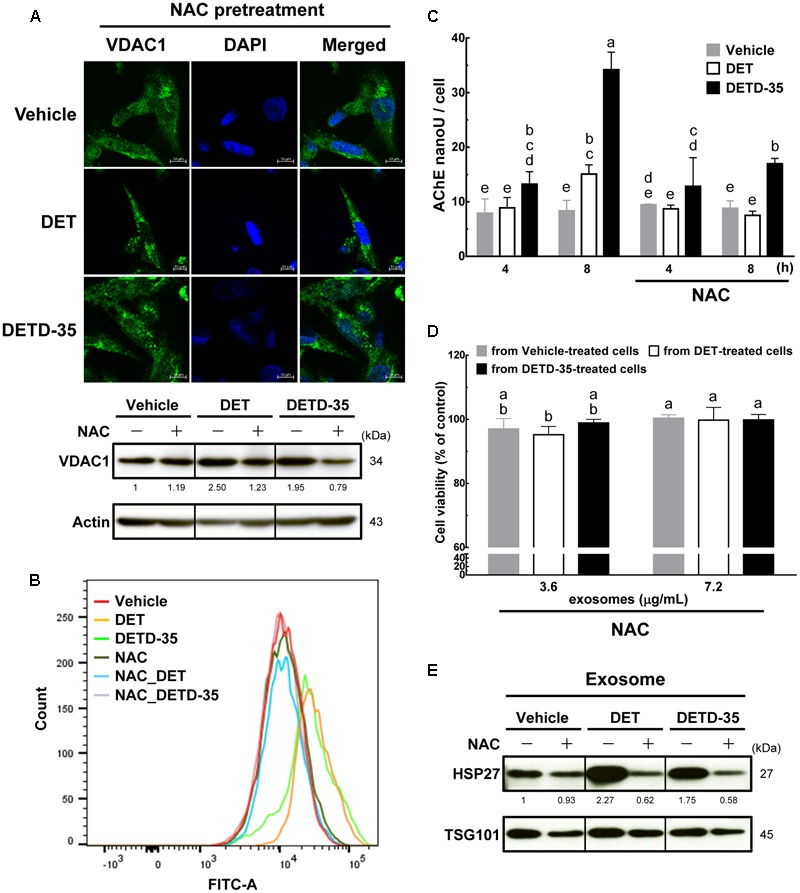
Pretreatment with ROS scavenger NAC prevents DET and DETD-35 induced mitochondrial dysfunction and reverted exosome release from cancer cells and their cytotoxic activity. **(A)** Immunofluorescence confocal micrographs of MDA-MB-231 cells pre-treated with 5 mM NAC for 1 h, and then treated with the vehicle (0.5% DMSO), 11 μM DET and 3 μM DETD-35 for 24 h. The treated cells were fixed using 100% ice-cold methanol, and stained with VDAC1 (green) and DAPI (blue) to visualize mitochondrial structures and nuclei. Immunoblotting of VDAC1 protein in the TNBC cells were pre-treated with 5 mM NAC for 1 h, and then treated with vehicle or compounds for 12 h. β-actin was used as a loading control. **(B)** MDA-MB-231 cells were pre-treated with 5 mM NAC for 1 h, and then treated with vehicle or either compound at the same concentration for 2 h. Cytosolic free calcium level in cancer cells was determined using Fluo-4 Ca^2+^ fluorescent dye using flow cytometry. **(C)** TNBC cells were pre-treated with 5 mM NAC for 1 h, and then treated with vehicle or either compound for 4 h and 8 h, and then the TNBC cell-secreted exosomes were collected from the culture media using differential centrifugation. Exosome samples were prepared by using a working solution containing PBS, RIPA buffer and protease inhibitors. The activity of acetylcholinesterase (AChE) was determined using fluorescence microplate reader. Data are mean ± SEM, *n* = 3. **(D)** Exosomes were isolated from TNBC cells pre-treated with NAC (5 mM) for 1 h, then treated with vehicle or either compound for 8 h; the culture media were collected to purify the exosomes. The purified exosomes (3.6 and 7.2 μg/mL, respectively) were added into the fresh-cultured MDA-MB-231 cells for 24 h, and the TNBC cell viability was determined using MTT assay. Different letters indicate significant differences (one-way ANOVA, *P* < 0.05). **(E)** The proteins isolated from exosomes prepared with the procedure described in **(D)** were subjected to immunoblotting of HSP27 and exosomal marker TSG101.

To further evaluate whether the ROS induced by either compound affects mitochondrial structures or functions, and/or TNBC cell-secreted exosome amounts, we used ROS scavenger, *N*-acetylcysteine (NAC) pretreatment for 1 h before either DET or DETD-35 treatment and then examined the mitochondrial structures by VDAC1 staining and confocal microscopy, and intracellular Ca^2+^ levels by Fluo-4 AM dye staining. Mitochondria-derived cytoplasmic vacuolation, up-regulated expression of VDAC1 protein (**Figure 7A**), and increased level of cytosolic free Ca^2+^ ions induced by both compound treatments (**Figure 7B**) were significantly attenuated by pretreatment with NAC. In addition, NAC pretreatment could also significantly reverse both compound-induced time-dependent release of exosomes from TNBC cells into culture media (**Figure 7C**); notably, the anti-proliferative activity of DET- and DETD-35-induced exosomes were abolished (**Figure 7D**). We have previously found that both DET and/or DETD-35 could significantly induce the generation of ROS in TS/A(ER+) mammary cancer cells, MDA-MB-231 cells or human BRAF mutant A375 melanoma cells (Lee and Shyur, 2012; Feng et al., 2016; Shiau et al., 2017). In the data about the exosomal proteome and the IPA toxicity list analysis, heat shock protein beta-1 (HSPB1), a member of the small heat shock protein (sHSP) family, also known as heat shock protein 27 (HSP27), and collagen alpha-2(IV) chain (COL4A2), a BM-related protein, were found correlated to the toxicity function of decrease of transmembrane potential of mitochondria. We further validated these results by western blotting, and observed that up-regulation of HSPB1 in compound responsive exosomes was significantly reduced by implementing NAC pretreatment (**Figure 7E**), suggesting that generation of ROS indeed plays a pivotal role as an upstream initiator to facilitate the cytotoxic effects of both DET and DETD-35 against TNBC cell activity by manipulating exosomal protein content and activity. Together, these results indicate that DET- or DETD-35-induced ROS is a crucial factor affecting the release and activity of secreted forms of exosomes from cancer cells and damaging the Ca^2+^ storage organelles, like mitochondria, in TNBC cells attributed to their anti-cancer effects.

## Discussion

A growing body of evidence indicates that exosomes mediate the delivery of proteins, mRNAs, and miRNAs from cancer cells to recipient or neighboring cells by cell-to-cell communication, which may assist in the creation of a metastatic niche and facilitate cancer cell progression and metastasis, or influence the activity and/or behaviors of recipient cells (O’Brien et al., 2013; Costa-Silva et al., 2015). Ca^2+^ ions are known to participate in the earlier stages of MVB biogenesis and the membrane fusion events regulating the secretion mechanism of exosomes (Savina et al., 2005; Urbanelli et al., 2013). In this study, we observed that the germacranolide sesquiterpene lactone DET and its derivative DETD-35 significantly induced the release of exosomes from MDA-MB-231 cells into media compared to the vehicle-treated cells, and a significant secretion flux was observed at 8-h treatment. The increase of exosome secretion in TNBC cells stimulated by DET or DETD-35 is likely the downstream cascade event after the compounds induce a significant amount of free Ca^2+^ ions in cell cytoplasm (observed within 2 h), because calcium chelator BAPTA-AM pretreatment significantly blockaded the ability of DET or DETD-35 to stimulate cancer cell release of exosomes. These results suggest the mechanisms by which DET and DETD-35 facilitate Ca^2+^-dependent exosome release from cancer cells.

The ER and mitochondria are two major calcium storage organelles. They might play a cooperative partnership role to maintain the balance of intracellular Ca^2+^ concentration when xenobiotic stimulation causes the release of ER Ca^2+^ into the cytosol through ER calcium-release channels (Mizuno et al., 2013). On the other hand, the mitochondria rapidly take up the increased cytosolic free Ca^2+^ by the mitochondrial metabolite transporter VDAC at the outer mitochondrial membrane and the mitochondrial calcium uniporter (MCU) at the inner mitochondrial membrane (Boland et al., 2013). Very recently, we have demonstrated that both DET and DETD-35 can significantly induce the formation of ER-derived cytoplasmic vacuoles resulting in paraptosis-like cell death in MDA-MB-231 cells within 24 h of treatment (Shiau et al., 2017). Here, we observed that shorter (2 h) and longer (24 h) treatment with both compounds could induce different degrees of mitochondria structural or functional damage in the TNBC cells, such as the loss of mitochondrial membrane potential within 2 h, over-regulated VDAC1 expression between 8 and 12 h, and formation of mitochondria-derived cytoplasmic vacuoles within 24 h (**Figure 1**). The results indicate that DET and DETD-35, aside from inducing ER damage, also elicited mitochondrial dysfunction in MDA-MB-231 cells. The mitochondrial dysfunction under chemical agent stimulation could be the result of changed mitochondrial permeability transition (MPT), increased ROS generation, damaged mitochondrial DNA (mtDNA) and mitochondrial respiration, and suppression of fatty acid oxidation (Vuda and Kamath, 2016). Besides, high concentrations of cytosolic Ca^2+^ and ROS are considered to be the regulators that enhance the opening of MPT pores (Nadanaciva and Will, 2009). We observed previously that DET induced ROS production within 2 h and facilitated ROS-associated ER stress in TS/A(ER+) mammary cancer cells (Lee and Shyur, 2012). In this and our previous studies (Shiau et al., 2017), we also detected DET- and DETD-35-induced ROS production within 2 h in MDA-MB-231 cells that we believe are associated with ROS-mediated ER stress probably including MPT pore opening, and subsequently promoting the release of ER or mitochondrial Ca^2+^ into the cytoplasm resulting in the enhancement of exosome secretion from the treated TNBC cells. Notably, all of the observed biological events triggered by DET/DETD-35 treatment in cancer cells can be reverted by pretreatment with ROS scavenger NAC, demonstrating that ROS induced by either compound plays a key role in their suppressive effects against TNBC cells.

Oxidative stress is known to regulate exosome secretion and their constituents. For example, oxidative stress influences exosomal mRNA composition that may protect recipient cells by strengthening their tolerance to oxidative stress and subsequent cell death (Eldh et al., 2010). In this study, we surprisingly observed that the exosomes collected from DET- and DETD-35-treated MDA-MB-231 cells have the ability to inhibit the same cancer cell proliferation. The yield of exosomes collected from ROS scavenger NAC plus DET or DETD-35 treated cells was significantly reduced, and the exosomes no longer showed anti-cancer cell activity. These data suggest oxidative stress induced by either compound might de-regulate exosome biogenesis and also their constituent compositions. By using a comparative MS-based quantitative proteomics approach coupled with Ingenuity Pathway Analysis (IPA) database analysis, we found that expression levels of BM-related proteins, such as laminin subunits alpha-2, alpha-5, and gamma-1, collagen alpha-1(V) chain, alpha-1(XII) chain, alpha-1(XVIII) chain, and alpha-2(IV) chain, and BM-specific heparin sulfate proteoglycan core protein, were down-regulated in TNBC cell-derived exosomes by either compound treatment. These proteins were reported to be involved in cancer cell adhesion, migration, proliferation, and differentiation, and affect tumor progression (Patarroyo et al., 2002). For example, laminin subunit alpha-5 fragments were able to bind with αvβ3 integrin to promote angiogenesis of endothelial cells (Gopal et al., 2016), and laminin-5 could interact with its receptor α6β4 integrin to promote cancer cell growth and survival (Zahir et al., 2003). Of note, a previous proteomic study showed that the expression of α6β4 integrin in TNBC-secreted exosomes was positively associated with lung metastasis of TNBC cells (Hoshino et al., 2015). Although we did not detect specific α6β4 integrin in our study, the down-regulation of laminin subunit alpha-5 responsive to DET or DETD-35 treatment may attenuate its interaction with integrin proteins and further abrogate TNBC cell proliferation, migration, lung metastasis among other activities we observed previously *in vitro* or *in vivo* (Nakagawa-Goto et al., 2016).

Epidermal growth factor-like repeat and discoidin I-like domain-containing protein 3 (EDIL-3) and lysyl oxidase homolog 2 (LOXL2) were reported to facilitate cancer cell migration and pre-metastatic niche formation which were also down-regulated in the DET or DETD-35 responsive exosomes. EDIL-3 could activate EGF receptor (EGFR) signaling to promote bladder cell migration (Beckham et al., 2014), and lysyl oxidase secreted by hypoxic breast tumor cells was considered to be a crucial mediator of pre-metastatic niche formation (Erler et al., 2009). The down-regulation of these proteins also supports, in part, the modes of action of DET or DETD-35 against TNBC cell activity.

On the basis of IPA toxicity list analysis, we may link compound-responsive proteins with biological mechanisms to accumulate more important information on the possible pharmacological response or potential mechanisms of DET or DETD-35 against TNBC cells. Intriguingly, a few compound-responsive exosomal proteins such as S100A9, S100A7, and glutamate-cysteine ligase modifier subunit (GCLM) related to oxidative stress, and HSPB1 involved in decrease of transmembrane potential of mitochondria were increased by DET/DETD-35 treatment. The S100 proteins are a multigenic family of calcium-modulated proteins, which interact with various receptors, and transcription factors among other target molecules involved in regulation of different biological processes (Donato et al., 2013). Our proteomic data show that the protein levels of S100A7 and S100A9 were upregulated in the exosomes collected from DET-/DETD-35-treated TNBC cells. Previous reports suggested that ROS production stimulates the expression of S100A7 or S100A9, and the regulation of the NF-κB pathway is involved in the induction of S100A7 (Carlsson et al., 2005). We have shown previously that DET deregulated the NF-κB/IKK signaling pathway known for its anti-inflammation as well as anti-cancer cell activities in macrophages or TS/A breast cancer cells. In addition, DET directly competes with DNA binding to NF-κB protein (Huang et al., 2010, 2013). We thus propose that up-regulation of S100A7 in exosomes originating from compound-treated TNBC cells might be associated with de-regulating NF-κB protein and the associated pathway, thus being involved in compound-induced anti-TNBC cell activity.

On the other hand, the up-regulation of compound-responsive exosomal protein heat shock protein beta-1 (HSPB1) can be reversed by ROS scavenger NAC pretreatment as validated by western blotting. Extracellular HSPB1 has been reported to have several functions, such as inducing cytokine production, modulating immune response, or promoting cell migration and proliferation by interacting with distinct receptors (Batulan et al., 2016). The overexpression of intracellular HSPB1 by transfecting HSPB1 expression vector in normal rat kidney (NRK)-52E cells was observed to promote the expression of autophagosomal marker protein microtubule-associated protein 1 light chain 3 (LC3) (Matsumoto et al., 2015). Very recently, we demonstrated that both DET and DETD-35 can promote paraptosis-like cell death in MDA-MB-231 cells through inducing the expression of LC3 and facilitating autophagosome accumulation (Shiau et al., 2017). We thus consider that up-regulation of HSPB1 in exosomes might be attributed to the anti-TNBC cell activity of DET and DETD-35.

In summary, this report shed light on the mechanisms of action of the plant derived sesquiterpene lactone compound DET and its derivative DETD-35 against TNBC cell activities. Induction of ROS/oxidative stress is a crucial factor that affects the release and activity of exosomes from cancer cells by altering protein composition and functions which suppress cancer cell viability. Whether or not the biogenesis of cancer cell-derived exosomes can also be altered by the plant compound treatment remains unclear and warrants further investigation.

## Author Contributions

J-YS, Y-QC, and L-FS conducted the conception and design of the experiments, acquisition of data, and analysis and interpretation of data. L-FS, KN-G, and K-HL provided the administrative, technical, or material support. All authors contributed to the development of methodology. J-YS and L-FS wrote and reviewed the manuscript. L-FS supervised the study.

## Conflict of Interest Statement

The authors declare that the research was conducted in the absence of any commercial or financial relationships that could be construed as a potential conflict of interest.
